# The Seventeenth Annual Meeting of the Central New York Homœopathic Medical Society

**Published:** 1883-09

**Authors:** C. P. Jennings

**Affiliations:** Secretary; Syracuse, N. Y.


### HOMŒOPATHIC PHYSICIAN,

A MONTHLY JOURNAL OF MEDICAL SCIENCE.

If our school ever gives up the strict inductive method of Hahnemann, we are lost, and deserve only to be mentioned as a caricature, in the history of medicine."—CONSTANTINE HERING.

Vol. III. SEPTEMBER, 1883. No. 9.

### THE SEVENTEENTH ANNUAL MEETING OF THE CENTRAL NEW YORK HOMŒOPATHIC MEDICAL SOCIETY.

SYRACUSE, N. Y., June 21st, 1883—10 A. M.

The Society met in the office of Wm. A. Hawley, M. D., A. E. Wallace, M. D., of Oneida, in the chair. Fourteen members were present.

The Secretary reported the sending to the A. I. H., through its President, a copy of the protest of this Society against its resolution on freedom of medical opinion.

Dr. Young reported the taking of the drug sent to him by the Secretary. Every time he took it, it was followed by aching in the calves of the legs, like the aching pains he suffered when sick with the ague in the West some years ago.

Dr. Wallace reported the taking of the same drug, a dose daily for forty days. Do not use coffee nor tea. Have muscular rheumatism. This was more severe during the taking of the drug. This aggravation may have been due to the cold winds prevailing at the time. The drug has not resulted in a cure.

Mrs. Dr. Emens took the same drug. Had no results.

Harlan P. Cole, M. D., of Bridgeport, Connecticut, formerly Professor of Anatomy in the Hahnemannian College, Chicago, Illinois, being present, was introduced to the Society by Dr. Hawley.

Being invited to address the Society, he exhibited a shoe for deformed feet, preferable to braces, because braces produce atrophy of the muscles. Where you can set the foot in proper position by your hand, the shoe will hold the foot in position while nature performs the cure. He has found in every case that has come into his hands, that the difficulty was in the tarsal region, and not in the malleolus.

Thanks were given to Dr. Cole.

Ordered, that the proving of the drug distributed by the Secretary be continued three months.

The Committee on publishing Dr. Hussey's report on Neural Analysis submitted, through Dr. Hawley, a statement: That Neural Analysis is a recent thing; that it is worthy of attention; that some published experiments will have to be revised; that a member of the Committee has found, by personal experiment, that the smallest quantity of sugar produces a marked effect; that this fact makes it necessary to ascertain with more exactness the effect of drugs; and therefore, time should be taken for further experiments and observations.

The statement of the Committee was accepted, and Committee continued.

On motion of Dr. Deuel, it was ordered that some of the members be appointed to prepare papers on a given subject for the next meeting.

On motion of Dr. Stowe, "The Essential Nature and Characteristics of Contagion and Infection" was chosen as the subject of said papers. Drs. Stowe, Deuel, and Harris were appointed to prepare papers on this subject for the next meeting.

A. E. Wallace, M. D., was elected president of this Society for the ensuing year; T. Dwight Stowe, M. D., vice-president, and C. P. Jennings, S. T. D., secretary and treasurer.

Dr. Hawley—At our last meeting I spoke of pain on moving or touching a limb as being a characteristic of Cina. Have had another clinical proof of this. A patient, suffering from inflammatory rheumatism, manifested this symptom. Cina cured her promptly. An adult.

Dr. Brewster—Had a case with the same symptom. Dysuria was present also, Cina cured.

Dr. Harris—Had some years ago a case similar to the one described by Dr. Hawley. A young woman. There was terrible itching of the vulva. Cina cured promptly. Great numbers of ascarides came away from the patient after the exhibition of Cina.

Dr. Stowe—A lady suffered from prosopalgia in left side of the face. Pallor, tongue coated a yellowish white, constipation, sweat on falling asleep at night, could not bear to be touched. Cina$^{200}$ in solution, one dose, cured her.

You will be interested in a recent surgical case. Last September a little boy was riding upon a load of boxes. The wagon ran over a stone. A box slid off, carrying the boy with it. He fell upon his head, fracturing the left parietal bone. I was called to him the third day after the accident. Found these symptoms: Coma; sterterous breathing; pulse irregular; wavering; vomiting; constipation; dilatation of pupils; insensibility to light, noise, and touch. Diagnosis compression of brain from extravasation. Advised an operation. Consent was given. Operated. There was no lesion of the scalp, but there was an excessive tumefaction. A large amount of coagulated blood flowed out, giving immediate relief. The pulse filled up, breathing became normal. The extravasation took place between the parietal bone and the *dura mater*. After a time secondary hemorrhage set in, tearing open the flaps, thus retarding recovery. About fifty days after the injury was received, exfoliation of the external plate of the parietal bone took place. The *spiculum* was drawn out, an incision having been made wide enough to admit of its passage. The boy is now well.

Adjourned. C. P. JENNINGS, *Secretary*.

